# Sustained Reduction in Severe Intraventricular Hemorrhage in Micropremature Infants: A Quality Improvement Intervention

**DOI:** 10.3390/children12030264

**Published:** 2025-02-21

**Authors:** Sabrina E. Wong, Lisa Sampson, Michael Dunn, Asaph Rolnitsky, Eugene Ng

**Affiliations:** 1 Sunnybrook Health Sciences Centre, Toronto, ON M4N 3M5, Canada; sabrina.wong@sunnybrook.ca (S.E.W.); lisa.sampson@sunnybrook.ca (L.S.); michael.dunn@sunnybrook.ca (M.D.); asaph.rolnitsky@sunnybrook.ca (A.R.); 2 Department of Paediatrics, University of Toronto, Toronto, ON M5S 1A1, Canada

**Keywords:** prematurity, intraventricular hemorrhage, brain injury

## Abstract

Background: Quality improvement (QI) interventions may reduce the incidence and severity of Intraventricular Hemorrhage (IVH) in the population of inborn micropremature infants (born at ≤26 weeks’ gestation) with the goal of improving outcomes in this high-risk population. Methods: A multidisciplinary team reviewed the current literature to develop a site-specific brain protective bundle. Baseline data were collected from June 2014 to February 2015, with interventions occurring from March 2015 to December 2015. The period of sustainability was assessed from January 2016 to December 2023. Control charts were used to analyze the effect of the interventions. Outcome measures included all grades of IVH, periventricular leukomalacia (PVL), necrotizing enterocolitis (NEC), and spontaneous intestinal perforations (SIP). Results: Brain care initiatives decrease the rate of severe IVH in the inborn micropremature infant population from a baseline of 21% to 6.45% with a sustained rate of 4.5% with no change to balancing measures. Conclusions: Brain-protective initiatives such as midline head positioning and minimal handling are associated with a significant and sustained reduction in severe IVH among inborn micropremature infants.

## 1. Introduction

Intraventricular hemorrhage (IVH) is a common serious complication of extreme prematurity and is even more prevalent in newborns less than 26 weeks of gestation. It is associated with neurodevelopmental complications such as intellectual impairment, cerebral palsy, and sensory–motor deficits. The incidence of severe IVH in 2015 in this inborn infant population at Sunnybrook Health Sciences Centre was 21%, higher than the median rates in national and international neonatal collaboration reports [1,2,3].

Extremely premature infants are at high risk for IVH and death [4]. With the evolution of neonatal-perinatal medicine, improvements in technology and knowledge have significantly improved the survival rates of infants born at less than 26 weeks’ gestation [5], frequently referred to as “micropremature” infants. IVH is traditionally categorized according to the stages published by Papile et al. (1978), with severe IVH being defined as intracranial hemorrhage that causes ventricular enlargement with blood in the ventricles and/or parenchymal injury or Papile’s stage 3 or 4 [6]. Due to the immaturity and fragility of the neonatal brain, micropremature infants are at high risk of severe IVH, which is strongly associated with adverse long-term neurodevelopmental outcomes and death [1,7,8].

Efforts to reduce neonatal morbidities have been published since the early 1990s. Preventative strategies and prophylactic treatments have been shown to reduce the rates of IVH in this vulnerable population [3,9,10,11,12]. These include antenatal corticosteroids [13], deferred cord clamping [14,15], and prophylactic indomethacin [3,9,13]. Research has also shown that optimizing management within the first 72 h of life, such as avoidance of hemodynamic fluctuations [3,9], minimal ventilation strategies [3,9], reduction in hypothermia [2,9,11,12,16,17], and head positioning [3,9,18], have been associated with a rate reduction in IVH [3,9,18,19].

Our Quality Improvement (QI) team has introduced several interventions to optimize brain care through the standardization and implementation of brain-protective strategies with an aim to decrease the rate of severe IVH in inborn micropremature infants from a baseline of 21% to 10% within 10 months. We focused on inborn micropremature infants because we targeted standardization of practices from the antenatal period up to the first 72 h of life. Within our regionalized health system, there are no means to standardize antenatal and delivery room practices in community health referring centers, so QI efforts to reduce severe IVH has minimal effect on the rate of IVH in outborn preterm infants (rate rates of outborn micropremature infants remained the same during the intervention period: baseline of 44% to 45% within 10 months).

## 2. Materials and Methods

This QI initiative was conducted at Sunnybrook Health Sciences Centre Neonatal Intensive Care Unit (NICU), a 48-bed, single-patient room, regional Level III academic perinatal center located in Toronto, Ontario, Canada. Our NICU is divided into 5 patient care areas—4 pods and a resuscitation room. We care for an average of 80 micropremature infants yearly, of whom 80% are inborn. Our unit is a member of the international Vermont-Oxford Network Collaborative [20] and the national Canadian Neonatal Network [21]. Both are large-scale NICU collaboratives that target QI in neonatal care by sharing knowledge, methods, and data and auditing and implementing QI methods to improve outcomes.

### 2.1. Population and Time Period

All inborn infants less than 26 weeks gestation were included in this initiative. Due to the unpredictable variability of practices at community hospitals and the potential deleterious effects of physical transfer by ambulance and/or planes between facilities, we decided to focus on the standardization of care for the inborn patient population with the knowledge that standardization could also improve outcomes for outborn patients. Infants who were outborn, had significant congenital abnormalities, were transferred out to a different hospital prior to the first head ultrasound, or were offered palliation within the first 72 h of life were not included in the analysis.

The pre-implementation period of 10 months was from 1 June 2014 to 28 February 2015. The intervention period was from 1 March 2015 to 31 December 2015. The assessment of sustainability was from 1 January 2016 through to 31 December 2023. The assessment of sustainability was initially assessed in three 10-month cycles: Cycle 1 from March 2015 to December 2015, Cycle 2 from January 2016 to October 2016, and Cycle 3 from November 2016 to August 2017. After this, assessment sustainability was further monitored on a yearly basis.

### 2.2. Planning

As part of a unit-based QI initiative to improve the outcomes of the micropremature infant population, a multidisciplinary steering committee including nurse practitioners, neonatologists, nurses, respiratory therapists, dieticians, pharmacists, educators, managers, and NICU parents was created. A subcommittee was formed in March 2014 with a special focus on the reduction in IVH and named the Brain Care Committee.

**Figure 1:**
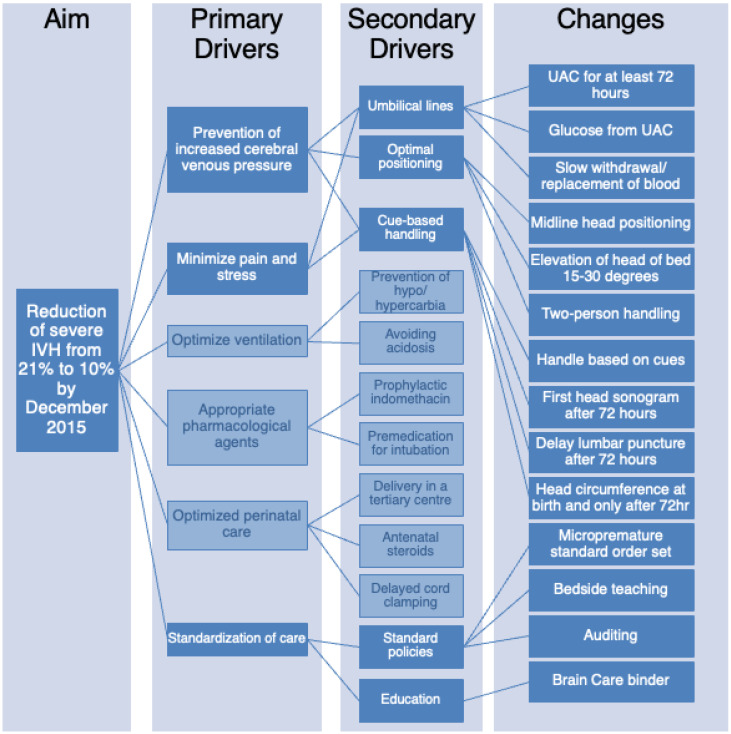
Reduction in IVH driver diagram showing the specific, measurable, applicable, realistic, and timely (SMART) aims.

The Brain Care Committee met monthly and reviewed all potentially better practices (PBP) for neuroprotection and developed a driver diagram of practices that may assist in the reduction in IVH based on the available literature evidence [3,9,12,18,19] (Figure 1). Six primary drivers were identified: (1) prevention of increased cerebral venous pressure, (2) minimization of pain and stress, (3) optimization of ventilation, (4) appropriate pharmacological agents, (5) optimized perinatal care, and (6) standardization of care. Upon review of the primary drivers, it was identified that drivers 3, 4, and 5 were already work in progress by other subcommittees. Thus, the focus of the Brain Care Committee narrowed to drivers 1, 2, and 6. Secondary drivers were created to guide the development of potential interventions. From there, changes in unit practice necessary to achieve the primary and secondary drivers were developed and implemented.

### 2.3. Interventions

In a review of the secondary drivers for the reduction in severe IVH, it was decided that five processes (highlighted in dark blue in Figure 1) would be the foci of this initiative—placing umbilical lines and maintaining them for a minimum of 72 h, exclusive use of an umbilical arterial catheter (UAC) for blood work including bedside glucose monitoring, optimal positioning by keeping head in midline and raising the head of the bed to 15–30 degrees, cue-based handling by limiting unnecessary procedures and handling including no weighing for 72 h, establishing standards of practice and education on the importance of brain protection practices. Several tests of changes using the plan-do-study-act (PDSA) methodology were planned to review the data and develop and implement the practice change.

#### 2.3.1. Creation of Standard of Care for the Micropremature Infant

A standard of care (SOC) was developed for potentially better neurodevelopmental care. This document titled the “SOC of the IVH Prevention in the Micropremature Infant” included new practices (these include the practices outlined in the Changes column in Figure 1) as well as reaffirmation of current practices (such as slow withdrawal of blood over 45 s from UAC, documentation of vital signs every hour, and orders for accurate fluid balance calculations) and that were adjusted to meet the new standard of practices for IVH prevention. Further, it clearly outlined each care action and included the rationale behind each new practice change. It was trialed with several new admissions over a two-month period (July–August 2014), modified based on feedback from the bedside staff, and finalized in November 2014.

#### 2.3.2. Creation of the Micropremature Infant Order Set

From bedside staff feedback, the SOC was felt to be cumbersome to work with as it included practices that were different from the standard NICU admission order set, leading to inadvertent omissions of certain practices. Checklists and standardized communication tools have been shown to reduce the variability of practice and improve compliance with guidelines [22]. So, to improve compliance with IVH prevention practices, especially in extremely low gestational age infants, the Micropremature Infant Admission Order Set was created to provide a systematic checklist of orders and quality improvement actions that needed to be carried out from birth through the first 72 h of age. Usability and functionality were tested on a convenient sample of ten admissions, and adjustments were made based on users’ feedback. The Micropremature Infant Order Set was formally approved as a standard of practice in June 2015.

#### 2.3.3. Implementation of Brain Care Initiatives: March 2015

The implementation of brain care initiatives brought about new changes to practice, specifically optimal positioning, minimal handling, and cue-based handling. For the purpose of this project, minimal handling was a term that included: handling the baby every 6 h (unless clinically indicated) to try to avoid disrupting the deep sleep state, all care or handling must have provided rationale documented, avoidance of unnecessary actions such as measuring the baby, avoidance of unnecessary head ultrasounds or echocardiograms, no painful or invasive procedures (such as lumbar punctures and PICC line placements), no use of a non-invasive blood pressure measurement with the use of a functioning UAC, and no kangaroo care for the first 72 h of life. Educational materials were adapted or developed to address each new practice. Specifically, the new Optimal Positioning Guidelines were developed, an identification card with the date and time of birth was created to place on each patient room door as a reminder of these brain care initiatives in the first 72 h, and an educational tip sheet was revised to include staff roles and responsibilities in this initiative. Multidisciplinary staff education was undertaken, and this information was subsequently incorporated into all new staff orientation sessions. A brain care education binder was created and placed in each patient area and in the resuscitation room for ease of reference. This binder included the selected literature for review and all the new policies and standards of practice. The brain care initiatives went live in March 2015 after 80% of the NICU staff had received the brain care education. Bedside audits were completed to assess compliance in June 2015, which identified areas of poor compliance, including elevation of the head of bed and two-person handling, for which follow-up PDSA cycles were developed to address these challenges.

#### 2.3.4. Elevation of the Head of the Bed

Poor compliance with the practice of head-of-bed elevation was related to practical reasons. It was felt that nursing a micropremature infant with ventilator tubing at that angle was difficult. To overcome that challenge, a PDSA cycle was developed, introducing visual instructional materials to help achieve the elevation of the head of the bed by 15–30 degrees and nursing tips on how to safely secure the infant during care. However, as bedside staff continued to struggle with maintaining a full 15–30 degree elevation, the protocol was modified to keep the head of the infant at any level above the body.

#### 2.3.5. Implementation of Two-Person Handling

Two-person handling has been shown to improve compliance in following brain protective strategies and allows for more comfort measures during handling times [9,12]. In April 2016, one year after implementing the brain care initiatives, two-person handling for all micropremature infants was rolled out within 3 cycles of change. The goal was to provide two-person care for 80% of stressful or painful interactions in the care of all micropremature infants. Bedside staffing ratios were adjusted to allow for either a second bedside nurse, a resource nurse, or a respiratory therapist to be available for handling times. Due to the significant change in care models, staffing allocation, and cost, this was implemented in three cycles: first with one baby only, second with an entire pod for one day shift (4 eligible infants), and third with an entire pod for a full 24 h (3 eligible infants). After the success of these 3 cycles, two-person handling was implemented as a standard of care. Once fully implemented, further education to include parents as a second person was initiated with the aim of a minimum of two interactions with parents and their infants per day.

#### 2.3.6. Implementation of the Small Baby Team/Program

Standardization of care has led to significant early improvements in the rate of severe IVH. Based on that principle, the small baby team model was developed in May 2017 to further substantiate the improvement in neurodevelopmental outcomes of micropremature infants, the most vulnerable population in the NICU. The model of a small baby unit where all micropremature infants are admitted to a dedicated pod within the NICU was unachievable in our unit from a logistical perspective. The alternative is to provide training to all staff to provide care for micropremature infants. Nurses with more than one year of NICU experience and all allied health professionals were asked to attend full-day education on the specific practices and the research evidence behind them. This education day included didactic learning, parents’ perspectives, as well as hands-on learning. The education helped to improve staff perceptions of the brain care initiatives with an aim to have micropremature infants cared for by a small baby team-trained provider for 80% of all care. This change in the model of care involved both an assessment of cost as well as a new staffing model. Over time the small baby team became the small baby program as the QI initiative evolved to encompass maternal and neonatal interventions across the entire Women and Babies Program. New NICU bedside nurses are provided general information about the small baby program at orientation and, based on previous experience or after one year, are invited to formally attend the small baby program training before qualifying to provide care to micropremature infants. Small baby program education is provided to new staff and trainees at their respective NICU orientation.

### 2.4. Measures and Definitions

Patients were identified prospectively until hospital discharge using the electronic medical record. Any inborn infant born less than 26 weeks’ gestation and admitted to the NICU was included. Measures not included in admission data were collected via retrospective chart review.

The primary outcome measure was the incidence of severe (Grade 3 or 4) IVH as diagnosed on head ultrasound (HUS) using Papile’s classification [6]. The first HUS was performed after 72 h as per the brain care initiative unless clinical condition and management require an earlier HUS. Hospital radiologists with expertise in neonatal HUS interpreted the ultrasounds, which were all reviewed subsequently by a neonatologist for accuracy and consistency of reporting. The secondary outcome of this study was mortality.

The primary process measures were adherence to the respective brain care initiatives—compliance with the standard of care of the micropremature infant and the micropremature infant order set, minimal handling and optimal positioning, and two-person handling. Balancing measures included all grades of IVH, cystic periventricular leukomalacia (PVL), and other global outcomes such as necrotizing enterocolitis (NEC), spontaneous intestinal perforation, and need for transfer for surgical consultation.

Analysis and interpretation of compliance with the proposed practices were performed using statistical process control charts with Microsoft Excel with QI Macros packages. Outcome measures were assessed using *p*-charts. For covariate-adjusted proportion comparisons, a generalized linear model was used, with *p*-values of <0.05 considered statistically significant.

## 3. Results

During the intervention period, 452 inborn micropremature infants were admitted to the NICU. The mean birth weight was 683 g with a mean gestational age of 24 + 4 weeks (Table 1 and Table 2).

**Figure 2:**
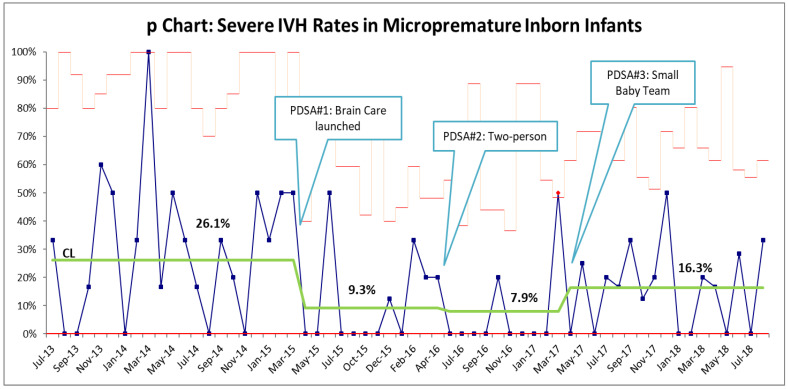
*p*-chart for the rate of severe IVH within the inborn micropremature population. The rate of severe IVH decreased from 21% to 10% within 10 months. PDSA #2 led to a further reduction to 6.25%, a rate that we have been unable to sustain.

Brain care initiatives were launched on 1 March 2015. Compliance with midline positioning guidelines as identified in bedside audits was 100% by the end of April 2015. Ten months after the launch of brain care initiatives, a statistically significant decrease in severe IVH rates from 21% to 6.45% was seen. By the end of December 2017, follow-up data showed a sustained statistically significant decrease of 10.7% (*p* = 0.001, 95% CI 0.08–0.29; Figure 2). Standardization of practices improved consistency of care and parental satisfaction despite a spike in census that tripled the number of births at borderline viability. Unit culture shift noted with improvement in IVH rates led to a culture that embraced change. In 2018, the rates of severe IVH were noted to plateau, and despite careful adherence to the brain care initiatives, the initial low rate of 6.5% could not be maintained. When reviewing the patient demographics over time, a shift in trend towards lower gestational age, especially an increase in 23-week infants, was noted. However, the increase in infants at the age of viability has not been sustained. While the inborn micropremature infant numbers remained below our historical means, the percentage of inborn to outborn micropremature infants has remained constant at around 20%.

**Figure 3:**
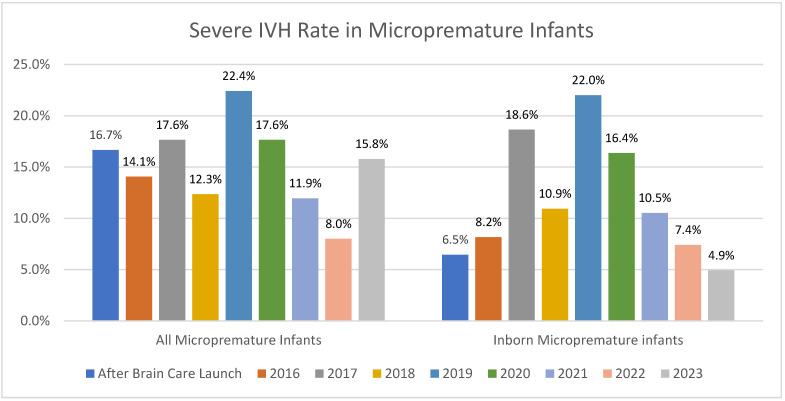
Yearly rates of IVH in both all micropremature admissions and in the inborn micropremature population.

While the number of inborn micropremature admissions may have decreased since 2019, so too has the rate of severe IVH with a decrease to 7.4% in 2022 and our lowest rate of 4.9% (Figure 3). It was noted that during an 11-month period (September 2023–August 2024), there was no severe IVH in our inborn premature (born less than 30 weeks) population.

## 4. Discussion

The implementation of neuroprotective brain care strategies for the care of inborn micropremature infants reduced our rate of severe IVH from 21% to a low of 4.9%. Practices such as minimal handling, midline positioning, two-person handling, and the creation of a specialized small baby program have all helped to sustain our improvements over the past 8 years.

The initial aim of focusing on the first 2 weeks of life was overwhelming to bedside staff, and the focus was narrowed to the first 72 h. The sheer number of changes included in the initial focus of change was not sustainable and were specifically narrowed to focus on midline positioning and cue-based handling, as these were the most widely accepted changes by the bedside staff. The inclusion of bedside staff in the development and education of new practice changes was crucial to their acceptance and successful implementation.

One of the most prominent pieces of feedback from staff was the need for standardization of practices, which led to the creation of the Micropremature infant order set. By ordering each brain-protective strategy at birth, it served as a checklist for staff to ensure that each target was being met and to be accountable for following each order. Standardization of practices further improved consistency of care and parental satisfaction despite a spike in census that tripled the number of births at borderline viability. Additionally, a unit culture shift was noted with the improvement in IVH rates, which has led to a culture that embraces change. All these noted changes are consistent with the literature that shows that care bundles [3,12] and changes towards cultures for improvement can improve care and outcomes [12].

The literature on the efficacy of brain care quality improvement strategies is conflicting. In 2021, a study by Persad et al. showed that there was no improvement in their rates of severe IVH when brain protective quality improvements were utilized. However, their study population included infants up to 30 weeks’ gestation. While the average birth weights were similar to this initiative, the average gestational age was higher by 4 weeks, which could indicate the larger impact on brain protective strategies in decreasing ages of viability. In 2024, a large systematic review by Edwards et al. showed that while quality improvement strategies reduced the rates of severe IVH, it was not consistent across all populations, with the reason for this inconsistency remaining unknown. Further, it is not clear as to which specific interventions had the greatest impact on rates of severe IVH [3].

It is possible that the standardization of care had the greatest impact on the rates of IVH. The literature has clearly shown that the standardization of tasks and communications tools has a large impact on improving health outcomes [22,23]. The continued process of auditing compliance to each new PDSA cycle allowed us to continually adjust and adapt to the needs of the ever-changing patient and staff populations.

A strength of this initiative was the multidisciplinary healthcare team. The inclusion of members of every discipline, including parents, allowed for a fully encompassing initiative where every member of the NICU was invested in the outcome. As a result, it led to a cultural change in the unit where the level of investment in quality improvement work was high. This allowed for active discussions and a desire to review each case when a severe IVH finding was identified. Staff found the process empowering, which led to more staff becoming involved and wanting to develop and lead initiatives within the larger project.

Limitations to this intervention include our patient population from a single-center academic Canadian hospital in a NICU that specializes in the care of extremely low birth weight premature infants, which could make these findings difficult to generalize to other settings. It is possible that the specific patient population (no surgical infants) allows for more consistency of practice and allows for a more seamless implementation of potentially better practices, some of which have not been found to be effective in other quality improvement studies or randomized control trials. As a quality improvement project, these data are further limited in the lack of collection of baseline information such as the use of antenatal steroids, mode of delivery, single vs. multiple births, or the correlation with typical neonatal outcomes other than IVH. Further, race and socioeconomic data were not collected, which further limits these findings.

## 5. Conclusions

The implementation of a standard order set and neuroprotective brain strategies such as minimal and cue-based handling was associated with a sustained reduction in the incidence of severe IVH in inborn micropremature infants.

## Figures and Tables

**Table 1:** Baseline demographics.

	Pre-Implementation	Cycle 1	Cycle 2	Cycle 3
Gestational Age (*n*)				
22 weeks	0	0	0	1
23 weeks	4	3	6	9
24 weeks	17	10	13	10
25 weeks	24	18	21	18
Total (*n*)	43	31	40	38
Avg (weeks)	25.58	24.4	24.38	24.18
Birth Weight (avg; grams)	736	741	688	690
Birth Weight (range; grams)	440–988	510–1025	395–990	374–1030
Sex, male (*n*)	21	16	18	21
Severe IVH (*n*)	9	2	4	0
Severe IVH (%)	20.9	6.5	10	0

Pre-implementation (June 2014–February 2015), Cycle 1 (March 2015–December 2015), Cycle 2 (January 2016–October 2016), Cycle 3 (November 2016–August 2017).

**Table 2:** Demographics based on the year.

	2016	2017	2018	2019	2020	2021	2022	2023
GA (weeks)								
22 weeks	0	1	3	2	1	3	3	3
23 weeks	8	21	18	14	14	14	9	5
24 weeks	19	12	15	18	20	16	19	22
25 weeks	28	25	28	16	20	23	23	28
Total (*n*)	56	59	64	50	55	56	54	58
BW (avg; grams)	736.37	719.64	684.28	669.44	654.75	660.61	685.17	661.41
Lowest	374	460	390	362	404	387	345	390
Highest	990	1030	980	1019	989	979	898	1065
Sex, male (*n*)	26	40	33	24	31	33	35	25
Severe IVH (*n*)	4	11	7	11	9	6	5	3

GA = gestational age, BW = birth weight.

## Data Availability

The original contributions presented in this study are included in the article. Further inquiries can be directed to the corresponding author.
